# General practice-related variation in oral anticoagulant treatment of atrial fibrillation: a nationwide cohort study

**DOI:** 10.3399/BJGPO.2024.0197

**Published:** 2025-05-07

**Authors:** Ina Grønkjær Laugesen, Claus Høstrup Vestergaard, Amanda Paust, Flemming Bro, Erik Lerkevang Grove, Anders Prior

**Affiliations:** 1 Research Unit for General Practice, Aarhus, Denmark; 2 Department of Public Health, Aarhus University, Aarhus, Denmark; 3 Department of Clinical Medicine, Aarhus University, Aarhus, Denmark; 4 Department of Cardiology, Aarhus University Hospital, Aarhus, Denmark

**Keywords:** family medicine, cardiovascular diseases, atrial fibrillation, prescribing, general practice

## Abstract

**Background:**

Guideline-adherent oral anticoagulant (OAC) treatment in atrial fibrillation (AF) remains a challenge. In Denmark, most patients with AF are treated in general practice. Nevertheless, determinants of OAC prescription in primary care are poorly understood.

**Aim:**

To investigate variation in OAC treatment adherence between general practice clinics and to identify clinic characteristics associated with a lower propensity to prescribe OAC treatment.

**Design & setting:**

Nationwide register-based cohort study including prevalent and incident patients with AF and CHA_2_DS_2_-VASc score ≥2 (*n* = 165 731) listed with Danish general practice clinics (*n* = 1666) in 2021.

**Method:**

The main outcome was OAC treatment adherence assessed as proportion of days covered (PDC). We used clinic OAC propensity to evaluate variation. OAC propensity was quantified as ratios between observed and expected adherence. Expected adherence was estimated based on the composition of the clinic patient populations. Sampled reference populations were constructed to account for random variation. Linear regression models examined associations between OAC propensity and clinic characteristics.

**Results:**

The PDC with OAC treatment in the AF population was 78%. OAC propensity in clinics in the 90^th^ percentile was 20% higher compared with clinics in the 10^th^ percentile; however, this difference was reduced to 3% when accounting for random variation. Modest associations were observed between clinic characteristics and OAC propensity. The most significant difference was in the correlation between geographic location and OAC propensity, showing an 8% gap between top and bottom-performing regions.

**Conclusion:**

The study suggests persistent underutilisation of OAC treatment in patients with AF and little variation in OAC prescription patterns across general practice clinics.

## How this fits in

In Denmark, most oral anticoagulant (OAC) treatment for patients with atrial fibrillation (AF) is prescribed by GPs, but little is known about their role in the persistent challenge of providing guideline-adherent treatment. In line with existing literature, this study finds substantial underuse of OAC treatment, yet very little variation was observed among general practice clinics in the propensity to prescribe OAC. This finding is surprising, as variation in clinical behaviour would be expected when treatment deviates from guideline recommendations. The study suggests a need to assess the reliability of register-based adherence measures and to investigate determinants of guideline adherence beyond general practice.

## Introduction

Atrial fibrillation (AF) affects millions of people worldwide, and a continuous rise in the prevalence is expected over the next decades.^1^ Patients with AF have a five-fold higher risk of stroke than the general population.^2^ Oral anticoagulant (OAC) treatment can effectively reduce this risk^3^ and is widely recommended in clinical guidelines for patients with AF.^4^ However, despite comprehensive evidence for the net benefit of OAC treatment in patients with AF, including vulnerable populations,^4^ adherence to OAC treatment guidelines remains a challenge.^5,6^

Previous research has highlighted the importance of patient-related, physician-related, and healthcare system-related factors for optimal OAC initiation and management.^7^ In Denmark, general practice serves as the primary entry point into the healthcare system and is pivotal in OAC management.^8,9^ Therefore, understanding the role of general practice is essential for optimising OAC in patients with AF.

Unwarranted variation, that is, variation in health care that cannot be explained by patient illness or preferences, may indicate potentially inappropriate clinical behaviour.^10^ In studies of variation, effective care is defined as the benefits of treatment far outweighing the risks, and treatment rates ideally approaching 100%.^10–12^ In the absence of contraindications, OAC treatment is recommended for all patients with AF with a CHA_2_DS_2_-VASc (congestive heart failure, hypertension, age ≥75  years, diabetes mellitus, prior stroke or transient ischaemic attack or thromboembolism, vascular disease, age 65–74  years, and female sex) score of ≥2.^4^ This means that OAC treatment can be considered effective care for these patients, and any variation in OAC use is most likely to be unwarranted.^10,11^ Accordingly, quantifying variation in OAC adherence among general practice clinics could provide valuable insights into the quality of care and identify opportunities for improvement.^13^ Knowledge on clinic characteristics associated with lower adherence to OAC guidelines may help tailor interventions to clinics with the greatest needs.

This study aimed to investigate the extent of variation in OAC treatment adherence in general practice and to identify clinic characteristics associated with a lower propensity to prescribe OAC.

## Method

### Design and population

We conducted a nationwide registry-based cohort study in Denmark from 1 January–31 December 2021.

We established an open cohort of prevalent and incident patients with AF and a CHA_2_DS_2_-VASc score of ≥2, meaning that all participants had an indication for OAC treatment according to guidelines.^4^ Participants were followed from the day they fulfilled the following inclusion criteria: aged ≥18 years, residing in Denmark for at least 5 consecutive years, listed with a Danish general practice clinic included in the study, AF diagnosis (International Classification of Diseases [ICD]-10: DI48*, including all sub-diagnoses) and a CHA_2_DS_2_-VASc score ≥2 (Supplementary Table S1), and no history of left atrial appendage closure procedure (procedure code: KFFW9*, including all subcategories). Patients were followed until the day they reached one of the following endpoints: undergoing a left atrial appendage closure procedure, no longer listed at a Danish general practice clinic included in the study, death, emigration, or end of study period, whichever came first.

To explore general practice-related variation, we identified all Danish general practice clinics in 2021. We excluded clinics with ≤500 listed patients on the first and last day in the study period and with a cumulative number of patient years of ≤500 in the study period. Clinics with <500 listed patients were considered abnormally small and were excluded to ensure reliable estimates.

### Data sources

The study used information from the Danish national health registers from 2001–2021.^14^ These data were linked at the individual level through the unique personal identification number in the Danish Civil Registration System, which also tracks vital status and migrations for all Danish residents.^15^

### Setting

The Danish tax-funded healthcare system provides residents with free and universal access to medical services. However, most specialised treatments require a referral, with GPs acting as gatekeepers.^9^ General practice operates with a list system, and more than 98% of the Danish population is listed with a general practice clinic. The lists comprise approximately 1600 patients per GP.^16^

### Outcome

The main outcome was OAC treatment adherence, defined as proportion of days covered (PDC)^17^ with OAC treatment. Adherence was assessed at the population level by comparing the total time covered by treatment with the total time at risk in the AF patient cohort.^18^ This approach provided a composite measure of the three phases of adherence: initiation, implementation, and persistence.

OAC encompassed drugs approved to prevent stroke and systemic embolism in AF, including vitamin K antagonists (VKAs; warfarin and phenprocoumon) and direct oral anticoagulants (DOACs; edoxaban, rivaroxaban, apixaban, and dabigatran).

For DOACs, the daily dose was calculated by multiplying the dose of the prescribed pill by the recommended number of daily dosages for the specific DOAC.

For VKAs, a model developed by Skeppholm and Friberg^19^ was utilised to assign a daily dosage to each prescription. This model predicts the daily dosage needed to achieve an international normalised ratio (INR) within the therapeutic range of 2.0–3.0, based on the patient’s sex and age at the time of prescription redemption. A factor 0.41 was used to convert warfarin to phenprocoumon dosages.^20^

The time covered by treatment was calculated at patient level using the refill gap method.^17^ The duration of each redeemed prescription was determined by dividing the dispensed medication amount by the estimated daily dosage. All prescriptions were given a grace period of 20%, that is, the duration of the prescription was extended by 20% beyond the dispensed amount. Additionally, a stockpiling interval of 30 days was permitted, allowing patients to carry over a maximum of 30-day supply of medication from one prescription to the next.

The PDC was calculated by dividing the aggregated time covered by redeemed OAC prescriptions by the total time at risk in the AF patient population during the study period.

We performed sensitivity analyses applying grace periods of 0% and 30% and allowing stockpiling for 0 and 60 days, respectively. None of these adjustments resulted in significant changes in OAC adherence.

### General practice clinic variables

General practice clinics were characterised based on size (clinic list size), composition of patient population (patient age, disease burden, and deprivation), and setting (city size and region) (Supplementary Table S2). The disease burden of each clinic was calculated using the Danish multimorbidity index (Supplementary Table S4),^21^ and patient deprivation score was calculated using a modified version of the Danish Deprivation Index (Supplementary Figure S1).^22,23^

### Statistical analyses

#### Variation in OAC adherence between clinics

To assess variation in OAC adherence between general practice clinics, we calculated the clinics’ propensity to prescribe OAC.^18^ The propensity score was defined as the observed adherence (observed PDC) in the clinic AF population divided by the expected adherence (expected PDC) in the clinic AF patient population (observed to expected [OE] ratio). To calculate the expected PDC, we used a Poisson regression model to predict the expected time covered by OAC for each patient when accounting for age, sex, socioeconomic status, ethnicity, and comorbidity (the included covariates are defined in Supplementary Tables S3 and S4). Using the clinics’ propensity score (OE ratio) when evaluating clinic OAC adherence accounted for differences in the clinics’ patient populations.

An OE ratio >1 indicated that the observed OAC treatment adherence in the AF population of the clinic was higher than expected based on the prediction model, and an OE ratio <1 suggested a lower-than-expected OAC treatment adherence.

The OAC propensity (OE ratio) of the clinics was ranked and depicted graphically, and the variation was quantified as the ratio between the 90^th^ percentile and the 10^th^ percentile of the OE ratio.^18,24^

To estimate the extent of variation that could be expected from chance alone (random variation), each clinic was assigned a reference AF population. These reference populations were constructed by substituting each patient with a matched patient from the patient cohort of other clinics by random sampling (matched by age, sex, and likelihood of receiving OAC predicted based on the remaining covariates defined in Supplementary Tables S3 and S4). The OE ratios for the reference populations were estimated and regarded as an approximation of the random variation.^18,24^

To express the variation exceeding random variation, the excess variation was quantified by dividing the 90^th^/10^th^ percentile ratio of the observed population by the 90^th^/10^th^ percentile ratio of the reference population.^18,24^

#### Association between clinic characteristics and adherence to guidelines on anticoagulant treatment of patients with AF

Linear regression analysis was used to explore the association between the characteristics of general practice clinics and their OAC propensity (OE ratio).

All analyses were performed in Stata (version 18)^25^ and reported according to the strengthening the reporting of observational studies in epidemiology (STROBE) guidelines.^26^

## Results

### OAC treatment coverage in the AF population

**Figure 1.**
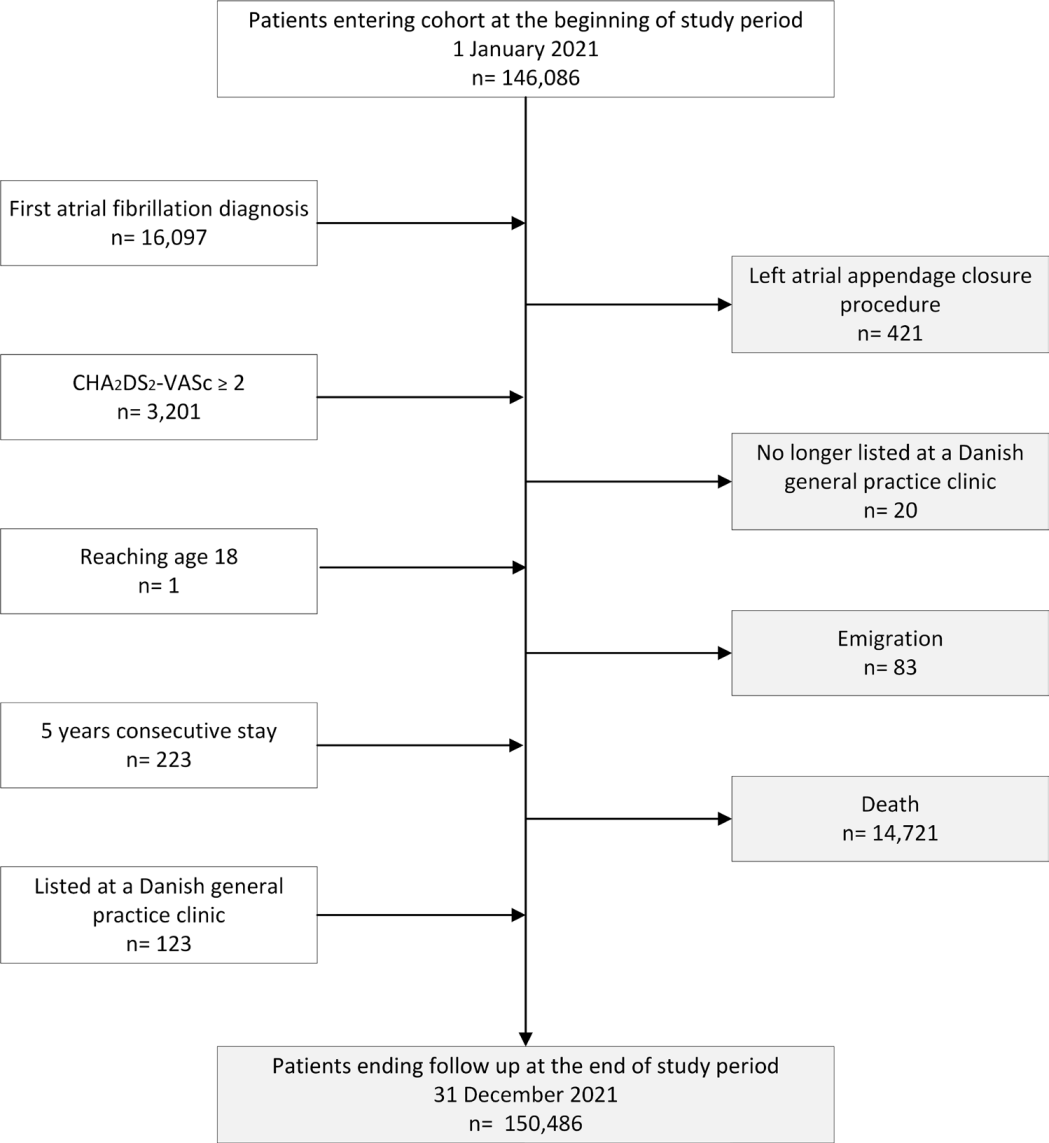
Flowchart illustrating formation of the patient cohort. Patients were included in the cohort on fulfilling all inclusion criteria: aged ≥18 years, residing in Denmark for at least 5 consecutive years, listed with a Danish general practice clinic included in the study, diagnosed with atrial fibrillation, CHA_2_DS_2_-VASc score≥2, and no history of undergoing a left atrial appendage closure procedure. During the study period, patients entered the cohort once all inclusion criteria were met. The last criterion fulfilled is depicted in the flowchart. Patients were followed until they reached one of the following endpoints: undergoing a left atrial appendage closure procedure, no longer being listed with a Danish general practice clinic included in the study, death, emigration, or the end of the study period, whichever came first. The white boxes indicate patients entering the cohort, and the grey boxes indicate patients leaving the cohort

From a total population of 4 533 350 million adult patients listed with GP clinics in Denmark, we included 165 731 patients with AF (Figure 1). The mean follow-up time was 324 days (standard deviation [SD] 92). The cohort represented 146,993 patient-years during the study period, of which 114,260 were covered by OAC treatment. This corresponds to an aggregated population-level PDC of 0.78, indicating an overall adherence rate of 78% among patients with AF

### Characteristics of general practice clinics

**Figure 2.**
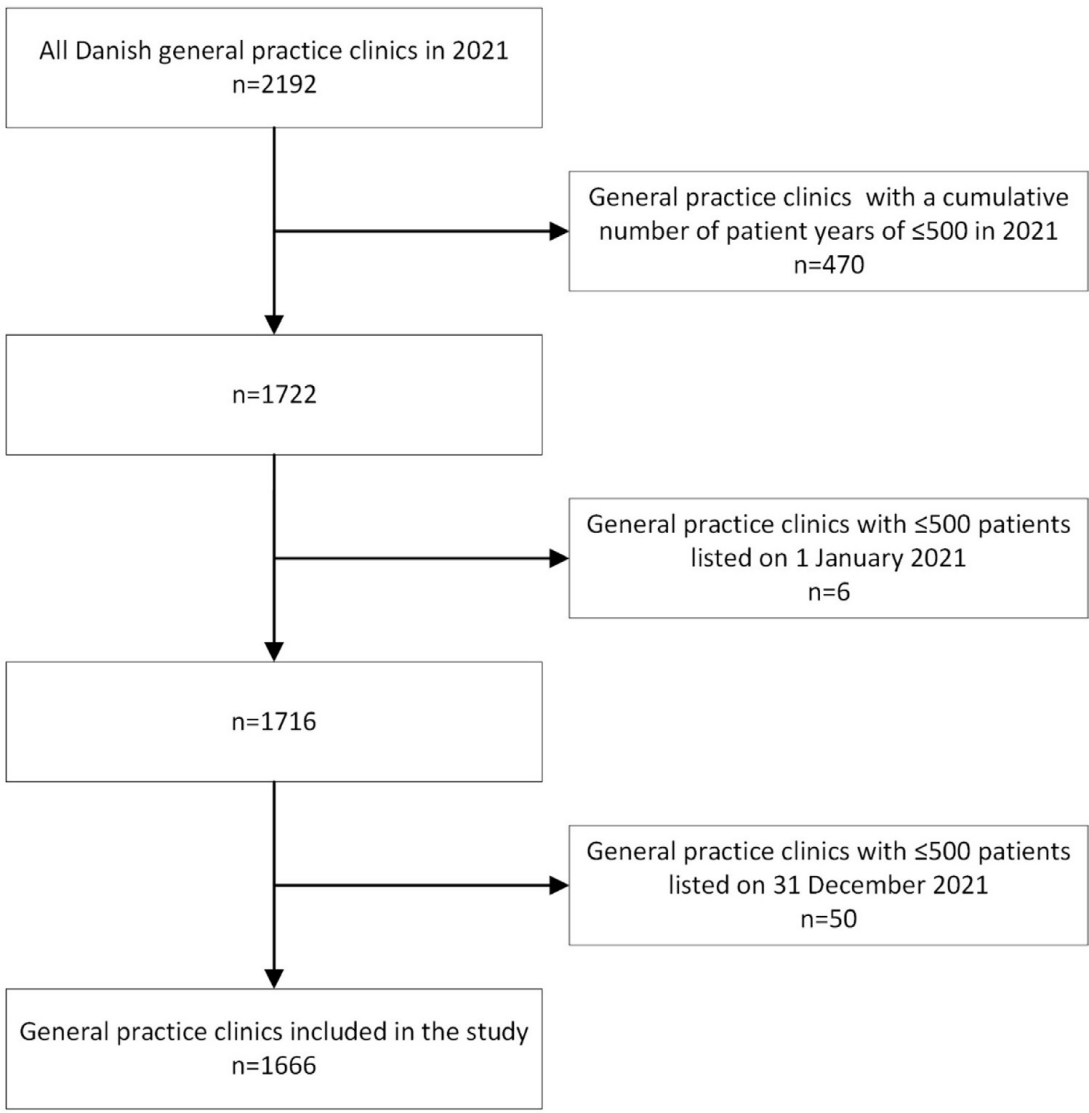
Flowchart illustrating the selection of participating general practice clinics. Based on a cohort of all adult Danish residents (Supplementary Table S2), all clinics with at least one patient listed in 2021 were identified. From the same cohort, the cumulative number of patient years, as well as the number of listed patients at the beginning and end of the study period were determined. Clinics with a cumulative number of patient years of ≤500 in 2021 or with ≤500 listed patients on 1 January or 31 December 2021 were excluded

We included 1666 general practice clinics (Figure 2). The clinics were characterised by size, patient population, and practice setting (Table 1).

**Table 1.** Demographic of general practice clinics

		General practice clinics, *n* (%)	AF patient years in 2021, years (%)
		Total 1666 (100%)	Total 146 993 (100%)
Size			
List size	<1500 patients	552 (33.1)	23 606 (16.1)
	1500–3499 patients	688 (41.3)	54 329 (37.0)
	≥3500	426 (25.6)	69 058 (47.0)
Patient population			
Average age	Low	556 (33.4)	29 491 (20.1)
	Average	555 (33.3)	56 389 (38.4)
	High	555 (33.3)	61 112 (41.6)
Disease burden	Low	556 (33.4)	30 685 (20.9)
	Average	555 (33.3)	55 585 (37.8)
	High	555 (33.3)	60 723 (41.3)
Deprivation	Affluent (index score <30)	513 (30.8)	44 390 (30.2)
	Average (index score 30–39)	709 (42.6)	69 487 (47.3)
	Deprived (index score ≥40)	444 (26.7)	33 116 (22.5)
Setting			
City size	<10 000 citizens	500 (30.0)	57 675 (39.2)
	10 000–99 999 citizens	538 (32.3)	52 272 (35.6)
	≥100 000 citizens	628 (37.7)	37 045 (25.2)
			
Region	North Denmark Region	154 (9.2)	16 049 (10.9)
	Central Denmark Region	343 (20.6)	31 973 (21.8)
	Region of Southern Denmark	344 (20.7)	34 049 (23.2)
	Capital Region of Denmark	595 (35.7)	40 077 (27.3)
	Region Zealand	230 (13.8)	24 846 (16.9)

AF = atrial fibrillation.

### Variation in OAC propensity among clinics

**Figure 3.**
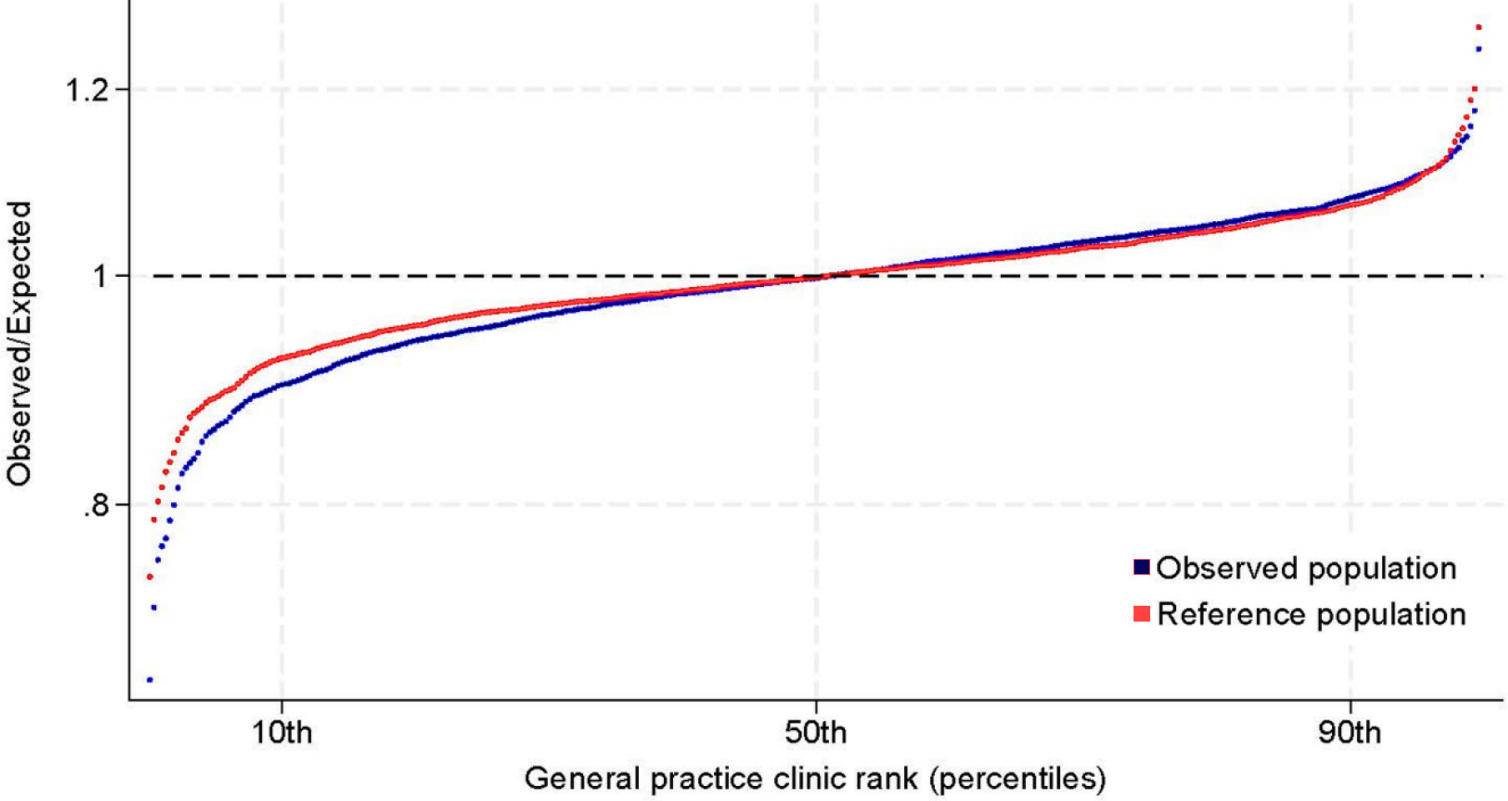
Variation in oral anticoagulant propensity between general practice clinics. Observed to expected ratios for adherence to oral anticoagulant treatment in general practice based on the actual patient population of the clinic (blue curve) and a reference population (red curve). Clinics were reported in groups of five on the plot to preserve anonymity

Figure 3 shows the graphical representation of the OE ratios in the clinics based on the observed and reference populations.

The extent of OAC propensity variation among clinics, measured as the ratio of the 90^th^ and 10^th^ percentile of the OE ratio, was 1.20, indication a 20 % higher OAC propensity in the clinicst at the 90^th^percentile. The variation in the sampled population (random variation) was 1.16 (16%), resulting in an excess variation of 1.03 (3%) (Table 2).

**Table 2.** Variation in oral anticoagulant propensity between general practice clinics

Observed variation (p90/p10)	Random variation (p90/p10)	Excess variation (observed/random)
1.08/0.90 = 1.20	1.07/0.92 = 1.16	1.20/1.16 = 1.03

p10 = 10^th^ percentile. p90 = 90^th^ percentile

### Association between clinic characteristics and OAC propensity

**Figure 4.**
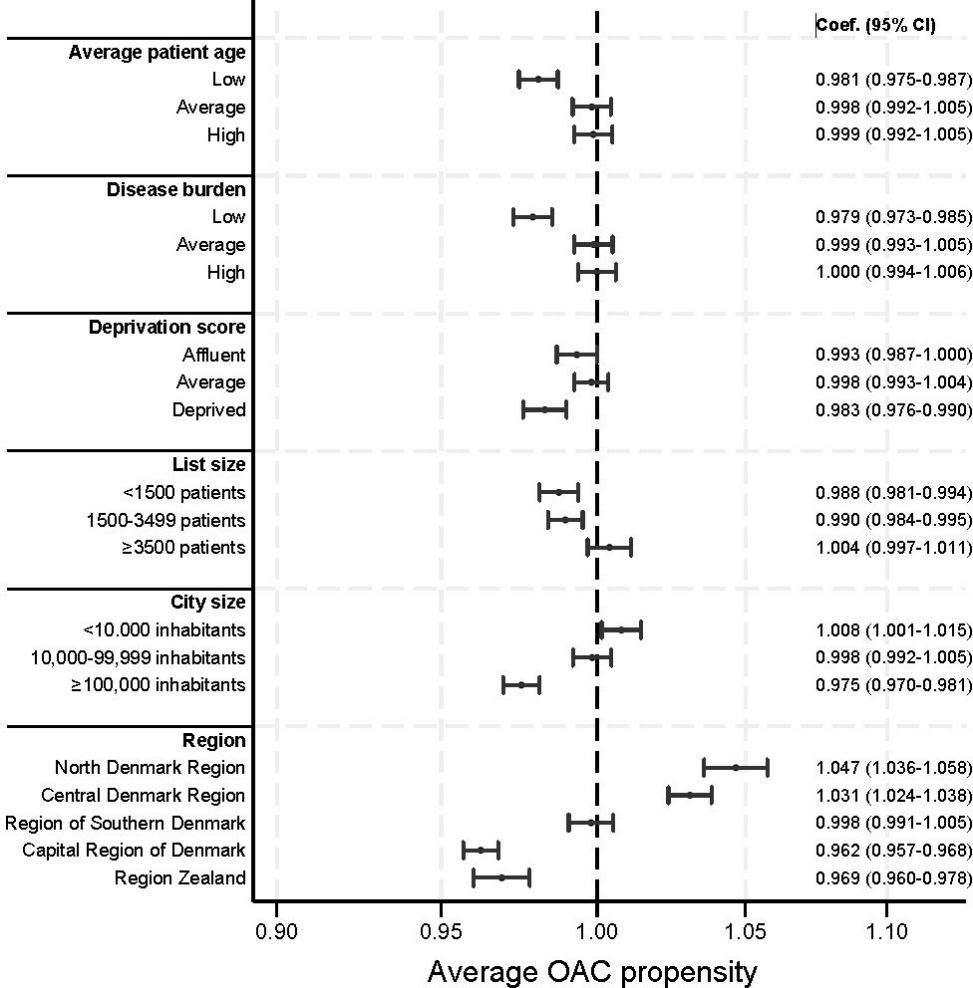
Association between general practice clinic characteristics and OAC propensity. Coef = coefficient. OAC = oral anticoagulant treatment

Figure 4 shows the average OAC propensity of the clinics. We found a decreased propensity to prescribe OACs in clinics serving patient populations with lower age (coefficient 0.981, 95% confidence interval [CI] = 0.975 to 0.987), fewer chronic diseases (0.979, 95% CI = 0.973 to 0.985), and higher deprivation score (0.983, 95% CI = 0.976 to 0.990). For practice organisation, we found lower OAC propensity in clinics with fewer patients, that is, in clinics with <1500 listed patients (0.988, 95% CI = 0.981 to 0.994) and 1500–3499 listed patients (0.990, 95% CI = 0.984 to 0.995). For practice setting, we found lower OAC propensity in clinics located in cities with ≥100 000 inhabitants (0.975, 95% CI = 0.970 to 0.981), in the Capital Region of Denmark (0.962, 95% CI = 0.957 to 0.968) and Region Zealand (0.969, 95% CI = 0.960 to 0.978), whereas clinics located in cities with <10 000 inhabitants (1.008, 95% CI = 1.001 to 1.015), in the North Denmark Region (1.047, 95% CI = 1.036 to 1.058) and the Central Denmark Region (1.031, 95% CI = 1.024 to 1.038) had increased propensity for OAC prescription. The largest difference in average OAC propensity scores was observed between clinics in the North Denmark Region and clinics in the Capital Region of Denmark, with OAC propensity being 8% higher in the North Denmark Region.

## Discussion

### Summary

We aimed to quantify OAC treatment adherence in the Danish AF population and employed variation methodology to explore the role of general practice in any guideline non-adherence. OAC treatment adherence measured by PDC was 78% in patients with AF and a moderate-to-high risk of ischaemic stroke, suggesting persistent underuse and possible guideline non-adherence. Yet, we found only limited variation in OAC prescription propensity across general practice clinics: a factor 1.20 difference in OAC propensity between practices in the 10^th^ and 90^th^ percentile, which was reduced to a factor 1.03 difference when the variation owing to chance alone was considered.

We observed a correlation between the geographical location of clinics and their propensity to prescribe OAC, as we found an 8% difference in average OAC prescription propensity rates between top and bottom-performing regions. Additionally, clinics with fewer listed patients, younger populations, fewer chronic diseases, higher deprivation scores, and urban settings tended to have lower OAC propensity. However, the effect sizes were modest, and the clinical significance may be limited.

### Strengths and limitations

We used comprehensive data from Danish national registers, which contain detailed information on health and sociodemographic characteristics of all participants, thereby strengthening our prediction model for expected OAC treatment adherence. Despite the comprehensive data available, the study was constrained by its reliance on redeemed prescriptions, lack of information on physician directives, and inability to distinguish between non-adherence owing to non-prescription by physicians and non-redemption of prescriptions by patients.

Detailed and robust data were available at the patient level, whereas little information was available on the structure, organisation, and demographic characteristics of clinics and GPs, thus limiting our investigations of the significance of these factors.

When assessing the variation between clinics, we considered the differences in patient population and the variation expected owing to chance, and this approach contributed to a robust and reliable method.

### Comparison with existing literature

The OAC treatment adherence of 78% found in our study population is consistent with findings in recent international meta-analyses.^5,6^ Our study included only patients with a CHA_2_DS_2_-VASc score of ≥2 to ensure OAC indication.^4^ Although some degree of non-adherence would be anticipated owing to contraindications, increased risk of bleeding, or patient preferences, we consider 22% of days without OAC coverage as a substantial gap in the adherence to current treatment guidelines.

Interestingly, we observed little variation in OAC prescription propensity among general practice clinics. The availability of pragmatic and practice-oriented guidelines is essential for achieving uniform and evidence-based treatment. In Denmark, a national clinical guideline on the treatment of AF is available at the Danish Society of Cardiology’s website (cardio.dk/af). This resource is widely available and is used within many specialties, including general practice. Additionally, the uniformly structured and publicly funded healthcare system may also promote consistent treatment practices.

However, the uniform performance in general practice is intriguing, as a certain amount of inter-practice variation in prescribing patterns would be expected when considerable guideline non-adherence is present.^10^

An important point to consider is whether the assessment of adherence reflects genuine under-treatment or if available data or methods lead to biased perspectives. The correct formation of the AF study population relies on accurate AF diagnoses and correct assessment of stroke risk. The AF diagnoses were extracted from the Danish National Patient Register, which offers data on hospital diagnoses and procedure codes.^14^ According to guidelines, all patients should be evaluated by a cardiologist when diagnosed with AF.^4^ In Denmark, the vast majority of these assessments are conducted in the public hospital setting, ensuring a high degree of data completeness for AF diagnoses. However, a positive predictive value of 92.6% has been reported for AF diagnoses recorded in the Danish registers.^27^ Therefore, some over-registration should be anticipated. Additionally, some uncertainty in the CHA_2_DS_2_-VASc scores is expected owing to the limitations of a register-based algorithm in predicting individual values. In our study, inaccuracies in both diagnoses and risk scoring would lead to inclusion of individuals without indication for OAC treatment in the AF patient cohort, which might entail potential bias towards underestimating OAC treatment adherence.

Another explanation for the low OAC treatment adherence in the population, but consistent performance in general practice, may be that OAC treatment adherence is primarily determined by factors that are either equally distributed among the clinics or are accounted for in the model calculating expected adherence and clinic OAC propensity. For example, patient characteristics such as socioeconomic status^28^ and cultural background^29^ are known to impact the likelihood of receiving OAC treatment. These were accounted for when assessing the variation between clinics, which could contribute to explain low adherence with minimal inter-practice variation. Additionally, in contrast to most studies on variation, our study estimated and accounted for random variation, which is a methodological approach that further contributed to the finding of minimal variation. Systemic structures and clinical behaviours at other levels of the healthcare system may also impact adherence. In our study, the geographical locations of the clinics appear to be correlated with OAC propensity, and this aligns with previous research.^30–32^ The Danish regions constitute the political and structural units responsible for hospital care and for health services provided by GPs and specialist practitioners.^33^ However, differences exist between regions in the organisation of patient pathways and supportive services such as pharmacological counselling, and IT systems. Such differences could impact treatment patterns as well as data collection and reporting. Moreover, a decreased OAC propensity was noted in urban areas, prompting further investigation into the underlying mechanisms of this observation.

### Implications for research and practice

This study identifies a remaining gap in adherence to OAC treatment guidelines in the Danish AF population, which should be addressed. However, we observed no systematic variation in general practice, which could indicate underlying disparities in clinical behaviour or organisational structures among clinics.^11,12^ In line with previous studies, we found that the propensity to prescribe OAC differs according to the regional location of the clinics, and further investigations should explore these regional disparities.

In conclusion, we found no significant variation in prescription patterns in general practice that could indicate opportunities for improving OAC treatment adherence in this setting. Our findings underscore the need to further clarify the extent of and the reasons for guideline non-adherence in OAC treatment for patients with AF. Such knowledge will be crucial for tailoring the content and addressing the best setting for interventions targeting OAC treatment adherence.

## Data

The data used in this study is available to authorised research institutions through collaborative agreements. Access to the Danish registers is granted by Statistics Denmark and the Danish Health Data Authorities. More information can be found on the website of Statistics Denmark (https://www.dst.dk/en/TilSalg/data-til-forskning).
